# When violence becomes endemic

**DOI:** 10.1007/s00038-017-1001-6

**Published:** 2017-08-03

**Authors:** Leslie F. Roberts

**Affiliations:** Columbia University Mailman School of Public Health, New York, USA

In 612 BC, an invading army reportedly diverted the Khosr River devastating one of the greatest cities of the world, Nineveh, killing large but unrecorded numbers in what was perhaps the first planned mass killing event of this kind. Similar, but more lethal intentional flooding events would follow in Central Asia and China, but 3000 years later; this attack is perhaps the most remembered event from the Kingdom of Assyria (Gleick 1994; Livius.org 2017). This tendency to remember with horror and clarity violent events may be inherent to human nature, but it is nearly the opposite of the collective message found in the pages of this edition. The overarching message from this body of work is that the 22 EMR countries are not advancing their health conditions as they should; and widespread, ingrained, diverse forms of intentional violence and its secondary effects are the primary reason why.

**Fig. 1:**
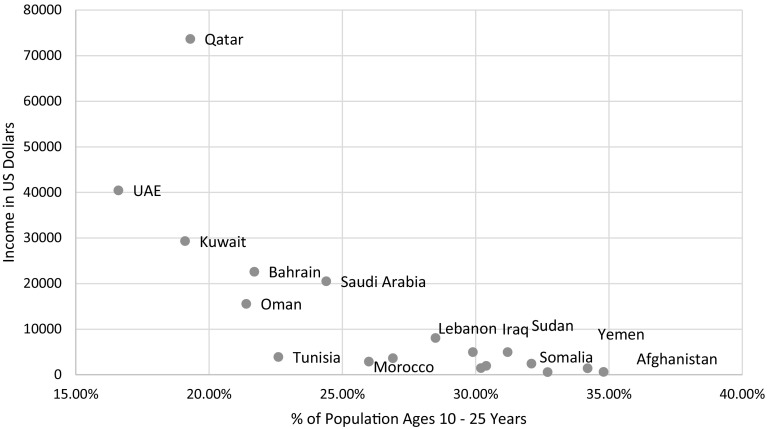
Income vs. %adolescents in Eastern Mediterranean region countries from child mortality collaborators, 2017

A large body of evidence suggests that low income and unemployed youths are both predictive of war and predictive of a country not being able to break out of a war cycle (Collier and Hoeffler 1998; Krause and Suzuki 2005). While income data were not available for four EMR countries including Syria, Fig. 1 displays an overwhelming trend of the most war-torn counties being disproportionately young and poor, and the most stable countries having fewer adolescents (Khalil and GBD 2015 EMR Adolescent Health Collaborators 2017). These data alone cannot tell us if instability causes high birth rates and low income, or if conditions of high birth rates and low income create conditions amenable to war. Yet the breadth of adverse health mechanisms described in this edition suggests the relationship is unquestionably complex.

In recent decades, there has been widespread appreciation of the global transition from death profiles dominated by infectious diseases to profiles dominated by non-infectious pathologies. What is less appreciated and made exceptionally clear from this body of work is how violence, and the associated compromised medical and social function, affects many different morbidities.

As stated by Dr. Khalil, the EMR has a rather even and exceptionally large span of health conditions, with under 5 mortality ranging from 112/1000 live births in Somalia and 89 in Afghanistan to 5.5 in UAE 6.2 in Bahrain (GBD 2015 EMR Child Mortality Collaborators 2017). Few will be surprised by the accompanying reports suggesting that diarrhea, maternal mortality, and respiratory disease all follow this same pattern with the highest rates in the war-torn countries (GBD 2015 EMR Diarrheal Disease Collaborators 2017; GBD 2015 EMR Lower Respiratory Infections Collaborators 2017; GBD 2015 EMR Maternal Mortality Collaborators 2017). What is more surprising are reports that Afghanistan had the highest rate of cardiovascular disease in 1990 and 2015 while Kuwait had the lowest in 1990 and Qatar in 2015 (GBD 2015 EMR Cardiovascular Disease Collaborators 2017). Khalil et al. report Afghanistan had the highest rates of traffic-related fatalities (GBD 2015 EMR Transportation Injuries Collaborators 2017), logically consistent with previously reported household survey data suggesting the 2003 invasion of Iraq corresponded with a dramatic increase in the traffic death rate there (Roberts et al. 2004).

What is most stunning in all of this information is Dr. Mokdad’s finding that war and legal violence across the region has gone up 850% over the past 15 years combined with Azzopardi et al. reporting that during this time, for both boys and girls, ages 10–14, 15–19, 20–24, war and legal violence has become the number one cause of death (GBD 2015 EMR Capstone Collaborators 2017; GBD 2015 EMR Adolescent Health Collaborators 2017). For girls of all ages and boys 10–14, it was not even among the top ten causes in 1990. Physical violence by firearms and the separate category of physical violence from other means have also moved into the top ten causes of death for boys 15–24, with all three categories of violence accounting for one-third of deaths in this demographic group.

Dr. Moradi-Lakeh’s article on intentional injuries, like previous assessments from the region speculates about the mechanisms by which violence degrades health and health care (GBD 2015 EMR Intentional Injuries Collaborators 2017; Webster 2016). Whatever the cause, the result is that curbing violence must now be the health community’s priority in the region. Brazen assaults on health, such as the pre-emptive invasion of Iraq, the using of poison gas on civilians in Syria, and the bombing of four hospitals in Aleppo on the same day in July 2016, have each been met without a UN resolution or any criminal prosecution (Al-Jazeera 2016; Bernard and Gordon 2017). The time for health professionals to cling to apolitical scientific neutrality has passed in the Eastern Mediterranean Region. After hundreds of medical facilities bombed and thousands of health workers selectively killed, we have figuratively become inured to Khors River type floods. It is likely only a sustained and vocal intolerance of such acts on the part of the health community, that results in public shaming and legal convictions, can curb this new endemicity of violence.
